# Efficacy and safety of camrelizumab plus transarterial chemoembolization in intermediate to advanced hepatocellular carcinoma patients: A prospective, multi-center, real-world study

**DOI:** 10.3389/fonc.2022.816198

**Published:** 2022-08-02

**Authors:** Ran You, Qingyu Xu, Qi Wang, Qingqiao Zhang, Weizhong Zhou, Chi Cao, Xiangzhong Huang, Honghai Ji, Penghua Lv, Hao Jiang, You Lu, Yong Jin, Yongjun Li, Long Cheng, Weidong Wang, Hao Xu, Xiaoli Zhu, Guowen Yin

**Affiliations:** ^1^ Interventional Radiology Department, Jiangsu Cancer Hospital and Jiangsu Institute of Cancer Research and The Affiliated Cancer Hospital of Nanjing Medical University, Nanjing, China; ^2^ Interventional Radiology Department, The First People’s Hospital of Changzhou, Changzhou, China; ^3^ Department of Obstetrics and Gynecology, The Affiliated Hospital of Xuzhou Medical University, Xuzhou, China; ^4^ Interventional Radiology Department, Jiangsu Province Hospital, Nanjing, China; ^5^ Interventional Radiology Department, Xuzhou Central Hospital, Xuzhou, China; ^6^ Interventional Radiology Department, Jiangyin People’s Hospital, Jiangyin, China; ^7^ Interventional Radiology Department, Yancheng No. 1 People’s Hospital, Yancheng, China; ^8^ Interventional Radiology Department, The Northern Jiangsu People’s Hospital, Yangzhou, China; ^9^ Interventional Radiology Department, The Second Affiliated Hospital of SooChow University, Suzhou, China; ^10^ Interventional Radiology Department, Nantong Tumor Hospital, Nantong, China; ^11^ Interventional Radiology Department, Wuxi People’s Hospital, Wuxi, China; ^12^ Interventional Radiology Department, The First Affiliated Hospital of SooChow University, Suzhou, China

**Keywords:** camrelizumab, transarterial chemoembolization, hepatocellular carcinoma, survival, adverse event

## Abstract

**Objective:**

Camrelizumab is a newly developed program-death receptor one inhibitor; the real-world evidence about its application in hepatocellular carcinoma (HCC) treatment is lacking. Therefore, this prospective, multi-center, real-world study evaluated the efficacy and safety of camrelizumab plus transarterial chemoembolization (TACE) in treating intermediate-to-advanced HCC patients.

**Methods:**

This study consecutively enrolled 101 intermediate to advanced HCC patients. All patients received camrelizumab-based treatment within 30 days of the perioperative period of the TACE operation. The primary outcome was progression-free survival (PFS), and the secondary effects were overall survival (OS), objective response rate (ORR), disease control rate (DCR), and AEs.

**Results:**

Specifically, the median PFS was 9.7 (95% confidence interval: 7.4–12.0) months, with a 1-year PFS rate of 30.6%. Meanwhile, the median OS was not reached (NR) yet, with a 1-year OS rate of 61.9%. Besides, the CR, PR, SD, and PD rates were 12.8%, 44.9%, 29.5%, and 12.8%, respectively. The ORR and DCR were 57.7% and 87.2%, respectively. More cycles of camrelizumab were independently correlated with prolonged PFS (hazard ratio (HR): 0.415, *P* = 0.002), whereas longer intervals between camrelizumab administration and TACE were independently associated with unfavorable PFS (HR: 1.873, *P* = 0.032). The incidence of total AEs was 90.1%; most AEs were grade 1 (20.8%), grade 2 (28.7%) and grade 3 (37.6%), while only 3 (3.0%) patients had grade 4 AEs.

**Conclusion:**

The camrelizumab plus TACE regimen is effective and safe, indicating its potential to serve as a promising treatment choice for intermediate to advanced HCC patients.

## Introduction

Hepatocellular carcinoma (HCC), one of the most common solid tumors, is the second leading cause of cancer-related deaths globally, with an estimated 830,180 new deaths in 2020 (1). Among these, about half of the HCC patients are derived from China. Meanwhile, more than 50% are diagnosed with intermediate to advanced HCC (2–5). Transarterial chemoembolization (TACE) is recommended for HCC patients with Barcelona Clinic Liver Cancer (BCLC) stage B who are not suitable for surgical resection, according to the guidelines issued by the American Association for the Study of Liver Diseases (AASLD) in 2018 (6). On the other hand, according to the Primary Liver Cancer Guidelines (2017 Edition) published in China, the TACE-based regimen is the primary treatment modality for HCC patients with China liver cancer (CNLC) stage IIb–IIIa (7). Even though TACE is one of the most common non-surgical treatments for patients with intermediate to advanced HCC, it can still lead to a post-therapy neoangiogenetic reaction or induce incomplete embolism, which further results in an unsatisfactory survival profile (7–9). Thus, exploring novel treatment choices in these patients should be highly prioritized.

Recently, TACE combined with other treatment modalities (including TACE plus tyrosine kinase inhibitors (TKIs) and TACE plus program-death receptor 1 (PD-1) inhibitor) is gradually becoming the primary regimen for patients with intermediate to advanced HCC, which has shown a good efficacy profile (10, 11). For instance, one study showed that TACE plus apatinib discloses a higher OS than TACE only in advanced HCC patients with macroscopic vascular invasion (median OS: 18.2 months vs. 8.5 months) (10). Another study indicated that TACE plus PD-1 inhibitor achieved an acceptable efficacy profile with a partial response (PR) of 22%, a stable disease (SD) of 78%, a 12-month progression-free survival (PFS) rate of 40%, and a 12-month overall survival (OS) rate of 71% (11). However, most of these studies are either single-armed or randomized controlled studies. In contrast, real-world studies remain rare, which might be more likely to reflect the actual clinical circumstances.

Camrelizumab, a PD-1 inhibitor, was independently developed by Jiangsu Hengrui Pharmaceuticals Co., Ltd. in China and has recently been approved by the Chinese Food and Drug Administration (CFDA) to treat hepatocellular carcinoma. A few studies have exhibited the efficacy of camrelizumab in patients with advanced HCC (12–14). For instance, a study disclosed that a combination of camrelizumab with sorafenib, TACE, and radiotherapy in treating advanced HCC patients with portal vein tumor thrombus achieved a median PFS of 15.7 months and a 1-year OS of 83.3% (12). Another study demonstrated that camrelizumab plus lenvatinib had a median PFS of 8.0 months in advanced HCC patients, which is higher than patients who received lenvatinib only (13). However, the sample size of these studies is relatively small. Besides, recent studies on the efficacy and safety of TACE plus camrelizumab in treating intermediate-to-advanced HCC patients are scarce.

Therefore, we conducted a prospective, real-world study with a large sample size (including 101 patients with intermediate to advanced HCC) and evaluated the efficacy and safety of camrelizumab plus TACE for treating patients with intermediate to advanced HCC.

## Methods

### Patients

This was a prospective, open-label, multi-center, single-armed, and observational real-world study. The study consecutively screened 101 intermediate to advanced HCC from 173 patients treated with camrelizumab plus TACE in 36 medical centers between August 2019 and March 2021. Patients who met the following conditions were eligible for enrollment: (i) diagnosis of primary HCC in line with Primary Liver Cancer Guidelines (2017 Edition) (7); (ii) over 18 years of age; (iii) BCLC stage B or C according to the criteria of 2018 version; (iv) with at least one measurable lesion as the target lesion revealed by contrast-enhanced computed tomography (CT) or magnetic resonance imaging (MRI) according to the modified Response Evaluation Criteria in Solid Tumors (mRECIST) criteria (15); (v) suitable for treatment with camrelizumab plus TACE; (vi) without serious abnormal blood, heart, lung, liver, or kidney function; and (vii) volunteered to participate in the study and willing to be followed up regularly. The patients with the following conditions were excluded: (i) had a contraindication to camrelizumab (an allergy to the active ingredient and excipients of camrelizumab). In detail, the active ingredient included camrelizumab (humanized anti-PD-1 monoclonal antibody); the excipients included, α, α-dihydrate trehalose, polysorbate 20, glacial acetic acid, sodium hydroxide, and water for injection); (ii) history of immunodeficiency disease or organ transplantation; (iii) concomitant with other cancers or malignancies; and (iv) pregnant or lactating women. The Institutional Review Board approved the current study with the approval number ChiECRCT20190186. All eligible patients provided written informed consent. This study was registered on the Chinese Clinical Trial Registry (available at: http://www.chictr.org.cn/) with the registration number ChiCTR1900026163.

### Treatment procedures

After enrollment, all patients received camrelizumab-based treatment within 30 days of the perioperative period of the TACE operation. The TACE operation was performed as described in a previous study (16). After identifying the tumor-feeding artery by visceral angiography, the microcatheter was catheterized by the distal super-selective method. Then, the chemotherapy drug solution of epirubicin mixed with lipiodol was slowly injected, followed by embolization using polyvinyl alcohol particles or gelatin sponge particles. The embolization ended when the contrast agent stagnated. During 30 days of the perioperative period of the TACE operation, camrelizumab was administered by intravenous drip at a dose of 200 mg for 30 min (between 20 and 60 min) each cycle, and every 2 weeks (Q2W) or every 3 weeks (Q3W) was a treatment cycle. Based on camrelizumab treatment, TKIs such as apatinib, lenvatinib, sorafenib, regorafenib, and anlotinib were also allowed for combination treatment. For the use of apatinib, it was recommended to stop the administration 4 to 7 days before TACE and start it on the day of the initiation of camrelizumab. Apatinib was administered orally at a dose of 250 mg daily. The camrelizumab-based treatment was continuously administered until the physicians determined that patients would not benefit from it anymore, the maximum duration of which was 2 years. Besides, the cycle of camrelizumab alone was also recorded, named as “cycles of camrelizumab.”

### Follow-up

The contrast-enhanced CT or MRI was examined at baseline and week 4 after the initiation of the treatment, then performed every 8 weeks, based on which the treatment response was assessed according to the mRECIST criteria (15), including complete response (CR), PR, SD, and progressive disease (PD). Adverse events (AEs) were closely monitored during the treatment, and the monitoring was continued up to the 28th day after the last administration of camrelizumab. The response was evaluated by the best overall response using the mRECIST criteria. Survival follow-up was performed monthly until the death of patients lost to follow-up or the end of the study, whichever came first, during which phone calls collected the survival data from all patients, their families, or local physicians, and the last date of follow-up was 1 July 2021.

### Outcome assessment

The primary outcome was PFS; the secondary effects were OS, objective response rate (ORR), disease control rate (DCR), and AEs. PFS was defined as the duration from the admission to the disease progression or death of patients, whichever came first; OS was defined as the duration from the enrollment to the death of the patient. The ORR was defined as the percentage of patients with CR or PR as the best response status; DCR was expressed as the percentage of patients with CR, PR, or SD as the best response status. The AEs were graded according to the National Cancer Institute Common Toxicity Criteria for Adverse Events (NCI-CTCAE) version 5.0 (17). Besides, data from patients with TACE treatment history were also extracted. These patients were classified as TACE refractory and not TACE refractory according to the criteria submitted by the Japan Society of Hepatology (18).

### Statistical analysis

SPSS 26.0 (IBM Corp., Armonk, New York, USA) and GraphPad Prism 7.01 (GraphPad Software Inc., San Diego, California, USA) were used for data analysis and figure construction, respectively. All 101 patients were included in the safety analysis, and 78 patients who underwent the same imaging examination (CT or MRI) as the baseline throughout the assessment process were included in the efficacy and survival analysis. Continuous data were presented as mean with standard deviation (SD), and categorial data were expressed as counts (percentage). Comparison between groups was evaluated by the chi-square test and Fisher’s exact test. The Kaplan–Meier method and log-rank test were applied to determine the difference in PFS/OS between groups. A Cox’s proportional hazard regression analysis was carried out for prognostic factor analysis, and a hazard ratio with a 95% confidence interval (CI) was shown. All significant variables (*P <*0.1 in univariate Cox’s regression analysis) were included in multivariate Cox’s regression for independent prognostic factor analysis. Statistical significance was derived if the two-sided *P*-value was less than 0.05.

## Results

### Study flow

**Figure 1:**
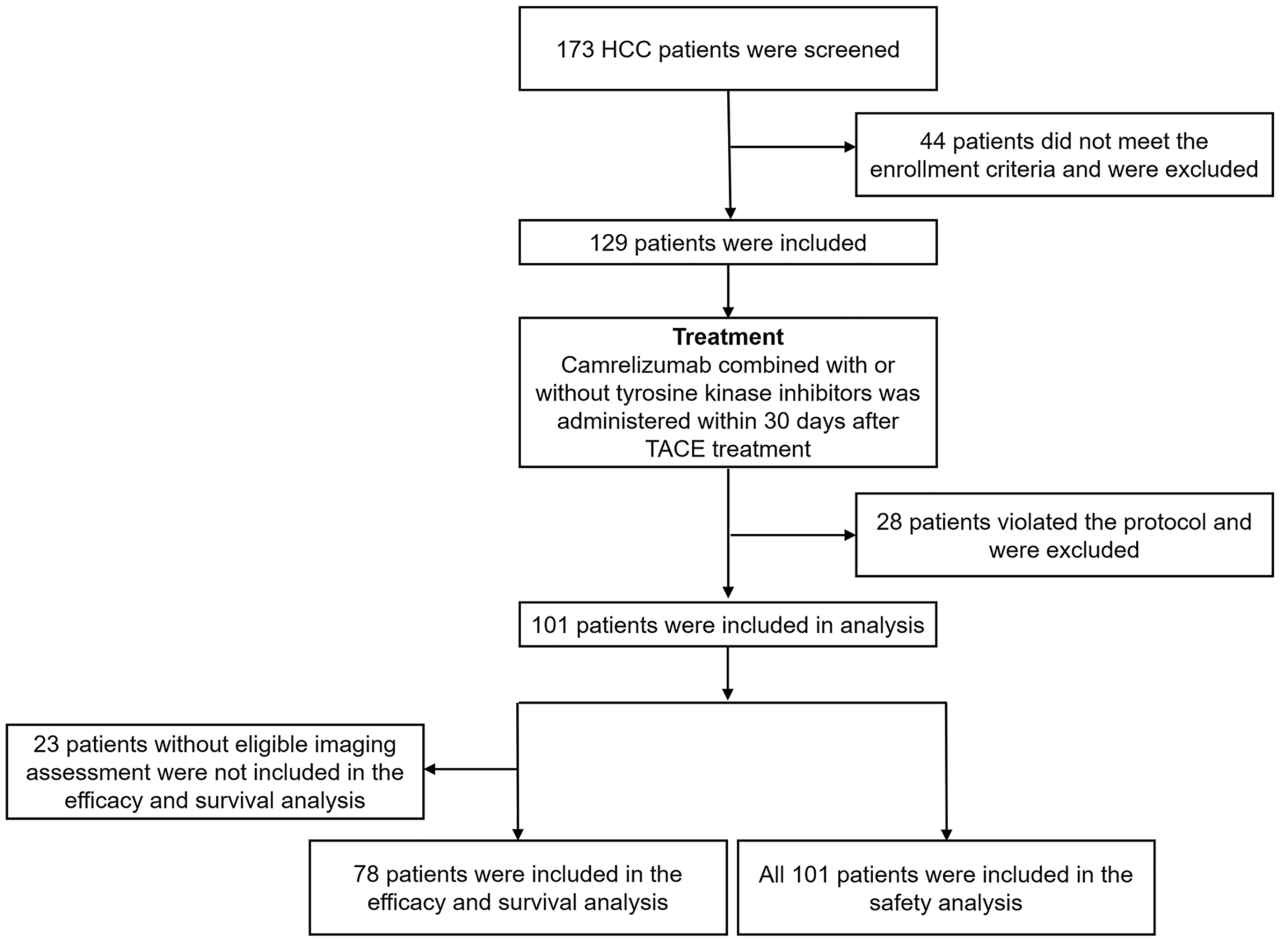
Study flow. HCC, hepatocellular carcinoma; TACE, transarterial chemoembolization.

Among the 173 HCC patients screened, 44 were excluded from this study because they did not meet the inclusion criteria; the remaining 129 patients were included. During the following treatment period, 28 patients were excluded for violating the study protocol. Subsequently, data from 101 patients were included in the analysis. Of these, the imaging results before and after treatment were inconsistent in 17 patients. Furthermore, imaging results from six patients were evaluated only at baseline but not after the treatment. Therefore, the efficacy and survival analysis excluded these 23 patients without eligible imaging assessment. Consequently, only 78 patients were included in the efficacy and survival analysis, and all 101 patients were included in the safety analysis. The detailed study flow is displayed in **Figure 1**.

### Clinical characteristics

The mean age of 101 enrolled HCC patients was 56.8 ± 11.2 years (**Table 1**), of whom 12 (11.9%) were females and 89 (88.1%) were males. In terms of the disease characteristics, 75 (74.3%) patients presented with hepatitis B virus positive; 1 (1.0%), 16 (15.8%), 26 (25.8%), and 56 (55.4%) patients exhibited CNLC stages of Ib, IIa, IIb, IIIa, and IIIb, respectively, while the CNLC stage of 1 (1.0%) patient was unknown (UK). Regarding the treatment history: 29 (28.7%) patients had never experienced TACE treatment before, while 32 (31.7%), 16 (15.8%), 7 (6.9%), and 17 (16.8%) patients had 1, 2, and 3, and more than three times of previous TACE treatments, respectively. Becauseof the current treatment in this study: 9 (8.9%) patients were administered with camrelizumab before TACE, whereas 92 (91.1%) patients received camrelizumab after TACE; Two (2.0%) patients received camrelizumab treatment every 2 weeks (Q2W), whereas 99 (98.0%) patients received camrelizumab treatment every 3 weeks (Q3W). Additionally, 84 (83.2%) patients were administered with camrelizumab within 7 days of the perioperative period of the TACE operation; nine (8.9%) patients received camrelizumab treatment within 8 to 14 days of the perioperative period of the TACE operation; and eight (7.9%) patients were treated with camrelizumab within 15 to 28 days of the perioperative period of the TACE operation. Furthermore, 48 (47.5%) patients received combination therapy with tyrosine kinase inhibitors (TKI), whereas 53 (52.5%) patients did not. Meanwhile, the most commonly used TKI was apatinib (26.7%), followed by lenvatinib (9.9%), sorafenib (4.0%), anlotinib (4.0%), and regorafenib (3.0%). Other detailed clinical characteristics are exhibited in **Table 1**.

**Table:** Table 1 Clinical characteristics.

Items	HCC patients (N = 101)
**Demographic characteristics**	
Age (years), mean ± SD	56.8 ± 11.2
Gender, No. (%)	
Female	12 (11.9)
Male	89 (88.1)
**Disease characteristics**	
HBV, No. (%)	75 (74.3)
ECOG PS score, No. (%)	
0	26 (25.7)
1	74 (73.3)
2	1 (1.0)
Child–Pugh class, No. (%)	
A	72 (71.3)
B	29 (28.7)
Extrahepatic metastasis, No. (%)	56 (55.4)
Vascular invasion, No. (%)	42 (41.6)
BCLC stage, No. (%)	
B	29 (28.7)
C	72 (71.3)
CNLC stage, No. (%)	
Ib	1 (1.0)
IIa	1 (1.0)
IIb	16 (15.8)
IIIa	26 (25.8)
IIIb	56 (55.4)
UK	1 (1.0)
AFP (ng/ml), No. (%)	
<400	57 (56.4)
≥400	39 (38.6)
UK	5 (5.0)
**Treatment history**	
Hepatectomy, No. (%)	27 (26.7)
Times of previous TACE, No. (%)	
0	29 (28.7)
1	32 (31.7)
2	16 (15.8)
3	7 (6.9)
>3	17 (16.8)
Refectory to TACE in patients with TACE treatment history, No. (%)	
No	26 (25.7)
Yes	31 (30.7)
Previous treatment lines, No. (%)	
First-line	82 (81.2)
Second-line	17 (16.8)
> Second-line	2 (2.0)
**Treatment in the study**	
Times of TACE, No. (%)	
≤3	88 (87.1)
>3	13 (12.9)
Timing of camrelizumab administration, No. (%)	
Before TACE	9 (8.9)
After TACE	92 (91.1)
Treatment cycle of camrelizumab	
Q2W	2 (2.0)
Q3W	99 (98.0)
Cycles of camrelizumab, No. (%)	
≤2	12 (11.9)
3–4	33 (32.7)
>4	56 (55.4)
Interval between TACE and camrelizumab administration, No. (%)	
Within 7 days	84 (83.2)
Within 8 to 14 days	9 (8.9)
Within 15 to 28 days	8 (7.9)
Treatment regimen, No. (%)	
Monotherapy of camrelizumab	53 (52.5)
Combination therapy with TKI	48 (47.5)
Apatinib	27 (26.7)
Lenvatinib	10 (9.9)
Sorafenib	4 (4.0)
Anlotinib	4 (4.0)
Regorafenib	3 (3.0)

HCC, hepatocellular carcinoma; SD, standard deviation; HBV, hepatitis B virus; ECOG PS, Eastern Cooperative Oncology Group Performance Status; BCLC, Barcelona Clinic Liver Cancer; CNLC, China liver cancer; UK, unknown; AFP, alpha-fetoprotein; TACE, transarterial chemoembolization; Q2W, every 2 weeks; Q3W, every 3 weeks; TKI, tyrosine kinase inhibitors.

### Clinical response

**Figure 2:**
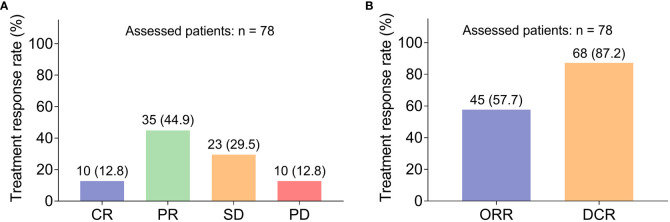
Treatment response. The CR, PR, SD, and PD rates in HCC patients receiving camrelizumab plus TACE **(A)**; The ORR and DCR rates in HCC patients receiving camrelizumab plus TACE **(B)**. HCC, hepatocellular carcinoma; TACE, transarterial chemoembolization; CR, complete response; PR, partial response; SD, stable disease; PD, progressive disease; ORR, objective response rate; DCR, disease control rate.

Specifically, 10 (12.8%) and 35 (44.9%) patients achieved CR and PR, respectively (**Figure 2A**). Besides, 23 (29.5%) patients retained SD, while 10 (12.8%) patients got PD. Thus, the ORR and DCR were 57.7% and 87.2%, separately (**Figure 2B**). Subgroup analysis disclosed that elevated cycles of camrelizumab were correlated with increased ORR (*P* = 0.023), while other clinical characteristics were not associated with the ORR or DCR (all *P >*0.050, **Table 2**). Additionally, images of two patients who achieved PR after the combination treatment were also shown (**Supplementary Figures 1**, **2**).

**Table 2:** Subgroup analysis of clinical response.

Items, No. (%)	ORR	Non-ORR	*P*-value	DCR	Non-DCR	*P*-value
Age			0.963			0.680
≤65 years	37 (57.8)	27 (42.2)		55 (85.9)	9 (14.1)	
>65 years	8 (57.1)	6 (42.9)		13 (92.9)	1 (7.1)	
Gender			1.000			1.000
Female	4 (57.1)	3 (42.9)		6 (85.7)	1 (14.3)	
Male	41 (57.7)	30 (42.3)		62 (87.3)	9 (12.7)	
HBV			0.224			0.107
No	12 (70.6)	5 (29.4)		17 (100.0)	0 (0.0)	
Yes	33 (54.1)	28 (45.9)		51 (83.6)	10 (16.4)	
ECOG PS score			0.673			0.348
0	13 (59.1)	9 (40.9)		21 (95.5)	1 (4.5)	
1	31 (56.4)	24 (43.6)		46 (83.6)	9 (16.4)	
2	1 (100.0)	0 (0.0)		1 (100.0)	0 (0.0)	
Child–Pugh class			0.608			1.000
A	35 (59.3)	24 (40.7)		51 (86.4)	8 (13.6)	
B	10 (52.6)	9 (47.4)		17 (89.5)	2 (10.5)	
Extrahepatic metastasis			0.522			0.172
No	21 (61.8)	13 (38.2)		32 (94.1)	2 (5.9)	
Yes	24 (54.5)	20 (45.5)		36 (81.8)	8 (18.2)	
Vascular invasion			0.630			0.086
No	27 (60.0)	18 (40.0)		42 (93.3)	3 (6.7)	
Yes	18 (54.5)	15 (45.5)		26 (78.8)	7 (21.2)	
BCLC stage			0.777			0.155
B	15 (60.0)	10 (40.0)		24 (96.0)	1 (4.0)	
C	30 (56.6)	23 (43.4)		44 (83.0)	9 (17.0)	
CNLC stage			0.788			0.518
Ib	1 (100.0)	0 (0.0)		1 (100.0)	0 (0.0)	
IIa	1 (100.0)	0 (0.0)		1 (100.0)	0 (0.0)	
IIb	7 (53.8)	6 (46.2)		13 (100.0)	0 (0.0)	
IIIa	11 (61.1)	7 (38.9)		16 (88.9)	2 (11.1)	
IIIb	25 (55.6)	20 (44.4)		37 (82.2)	8 (17.8)	
AFP			0.070			0.290
<400 ng/ml	31 (66.0)	16 (34.0)		43 (91.5)	4 (8.5)	
≥400 ng/ml	13 (44.8)	16 (55.2)		24 (82.8)	5 (17.2)	
UK	1 (50.0)	1 (50.0)		1 (50.0)	1 (50.0)	
Hepatectomy			0.384			1.000
No	30 (54.5)	25 (45.5)		48 (87.3)	7 (12.7)	
Yes	15 (65.2)	8 (34.8)		20 (87.0)	3 (13.0)	
Times of previous TACE			0.617			0.490
0	12 (57.1)	9 (42.9)		18 (85.7)	3 (14.3)	
1	16 (61.5)	10 (38.5)		23 (88.5)	3 (11.5)	
2	9 (64.3)	5 (35.7)		13 (92.9)	1 (7.1)	
3	4 (66.7)	2 (33.3)		6 (100.0)	0 (0.0)	
>3	4 (36.4)	7 (63.6)		8 (72.7)	3 (27.3)	
Previous treatment lines			0.577			0.538
First-line	37 (58.7)	26 (41.3)		56 (88.9)	7 (11.1)	
Second-line	7 (50.0)	7 (50.0)		11 (78.6)	3 (21.4)	
> Second-line	1 (100.0)	0 (0.0)		1 (100.0)	0 (0.0)	
Times of TACE, No. (%)			0.356			0.199
≤3	36 (55.4)	29 (44.6)		55 (84.6)	10 (15.4)	
>3	9 (69.2)	4 (30.8)		13 (100.0)	0 (0.0)	
Timing of camrelizumab administration, No. (%)			1.000			0.574
Before TACE	4 (66.7)	2 (33.3)		5 (83.3)	1 (16.7)	
After TACE	41 (56.9)	31 (43.1)		63 (87.5)	9 (12.5)	
Treatment cycle of camrelizumab			0.176			1.000
Q2W	0 (0.0)	2 (100.0)		2 (100.0)	0 (0.0)	
Q3W	45 (59.2)	31 (40.8)		66 (86.8)	10 (13.2)	
Cycles of camrelizumab, No. (%)			**0.023**			0.080
≤2	2 (25.0)	6 (75.0)		8 (100.0)	0 (0.0)	
3–4	11 (45.8)	13 (54.2)		18 (75.0)	6 (25.0)	
>4	32 (69.6)	14 (30.4)		42 (91.3)	4 (8.7)	
Interval between TACE and camrelizumab administration			0.454			0.658
Within 7 days	40 (59.7)	27 (40.3)		58 (86.6)	9 (13.4)	
Within 8 to 14 days	3 (60.0)	2 (40.0)		5 (100.0)	0 (0.0)	
Within 15 to 28 days	2 (33.3)	4 (66.7)		5 (83.3)	1 (16.7)	
Treatment regimen			0.341			1.000
Monotherapy of camrelizumab	21 (52.5)	19 (47.5)		35 (87.5)	5 (12.5)	
Combination therapy with TKI	24 (63.2)	14 (36.8)		33 (86.8)	5 (13.2)	

ORR, objective response rate; DCR, disease control rate; HBV, hepatitis B virus; ECOG PS, Eastern Cooperative Oncology Group Performance Status; BCLC, Barcelona Clinic Liver Cancer; CNLC, China liver cancer; AFP, alpha-fetoprotein; UK, unknown; TACE, transarterial chemoembolization; Q2W, every 2 weeks; Q3W, every 3 weeks; TKI, tyrosine kinase inhibitors. The bold values indicate the comparison with statistical significance.

### Survival profiles

**Figure 3:**
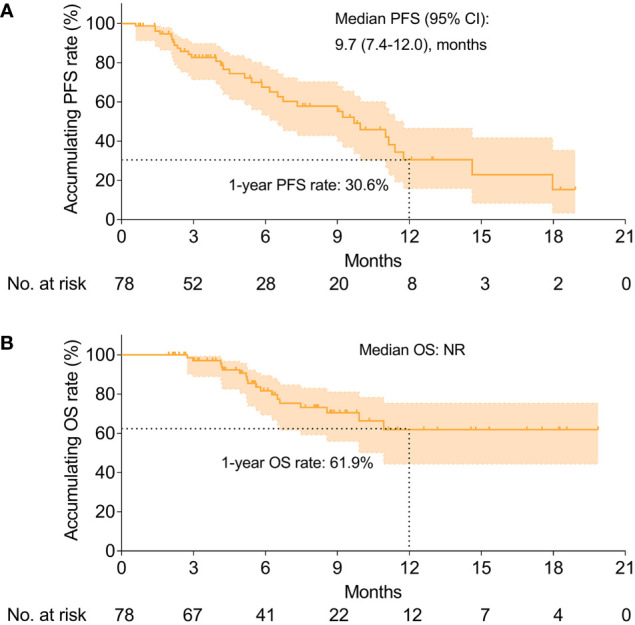
Survival profiles. The PFS **(A)** and OS **(B)** in HCC patients receiving camrelizumab plus TACE. HCC, hepatocellular carcinoma; TACE, transarterial chemoembolization; PFS, progression-free survival; OS, overall survival; NR, not reached.

The median PFS was 9.7 (95% CI: 7.4–12.0) months, with a 1-year PFS rate of 30.6% (**Figure 3A**); Besides, the median OS was not yet reached (NR), with a 1-year OS rate of 61.9% (**Figure 3B**).

Subgroup analysis of PFS revealed that higher cycles of camrelizumab were associated with the favorable PFS (hazard ratio (HR): 0.503, 95% CI: 0.320–0.792, *P* = 0.003, **Table 3**); a longer interval between camrelizumab administration and TACE was related to the unfavorable PFS (HR: 1.702, 95% CI: 1.039–2.790, *P* = 0.035), whereas other clinical characteristics were not associated with the PFS (all *P >*0.05). In terms of the subgroup analysis of OS, it revealed that the presence of vascular invasion (HR: 4.152, 95% CI: 1.476–11.680, *P* = 0.007), more times of previous TACE (HR: 1.537, 95% CI: 1.110–2.149, *P* = 0.012) and a longer interval between camrelizumab administration and TACE (HR: 2.542, 95% CI: 1.398–4.620, *P* = 0.002) were associated with declined OS, whereas higher cycles of camrelizumab were associated with favorable OS (HR: 0.401, 95% CI: 0.221–0.729, *P* = 0.003).

**Table 3:** Subgroup analysis of PFS and OS.

Items	PFS			OS		
	Median PFS (95% CI), months	HR (95% CI)	*P*-value	1-year OS rate, (%)^*^	HR (95% CI)	*P*-value
Age			0.473			0.848
≤65 years	10.0 (7.3–12.6)	1.000		65.4	1.000	
>65 years	9.0 (4.5–13.5)	1.393 (0.563–3.445)		48.5	1.116 (0.365–3.409)	
Gender			0.832			0.856
Female	incalculable	1.000		71.4	1.000	
Male	9.7 (7.4–12.0)	0.878 (0.264–2.924)		61.0	0.873 (0.200–3.805)	
HBV			0.220			0.290
No	18.0 (incalculable)	1.000		88.9	1.000	
Yes	9.2 (5.9–12.5)	2.113 (0.639–6.992)		58.2	2.976 (0.395–22.402)	
ECOG PS score		0.926 (0.474–1.810)^#^	0.822		1.223 (0.501–2.984)^#^	0.659
0	7.3 (3.0–11.6)	–		63.8	–	
1	10.0 (7.7–12.2)	–		61.8	–	
2	incalculable	–		incalculable	–	
Child–Pugh class			0.118			0.605
A	9.0 (5.7–12.3)	1.000		56.9	1.000	
B	18.0 (6.0–30.0)	0.488 (0.199–1.199)		74.7	0.745 (0.244–2.271)	
Extrahepatic metastasis			0.509			0.143
No	11.0 (4.7–17.3)	1.000		68.8	1.000	
Yes	9.2 (5.3–13.2)	1.265 (0.630–2.537)		58.0	2.093 (0.778–5.628)	
Vascular invasion			0.170			**0.007**
No	11.0 (8.2–13.8)	1.000		76.3	1.000	
Yes	6.2 (4.4–8.0)	1.627 (0.811–3.263)		43.7	4.152 (1.476–11.680)	
BCLC stage			0.685			0.138
B	9.7 (7.0–12.4)	1.000		74.7	1.000	
C	10.0 (4.4–15.5)	1.174 (0.540–2.553)		56.7	2.563 (0.739–8.887)	
CNLC stage		1.220 (0.832–1.790)^#^	0.308		1.852 (0.960–3.569)^#^	0.066
Ib	incalculable	–		incalculable	–	
IIa	11.0 (incalculable)	–		incalculable	–	
IIb	9.7 (6.2–13.2)	–		80.0	–	
IIIa	11.8 (0.5–23.0)	–		49.8	–	
IIIb	9.2 (5.2–13.2)	–		59.6	–	
AFP			0.255			0.090
<400 ng/ml	9.0 (5.5–12.5)	1.000		71.5	1.000	
≥400 ng/ml	11.4 (8.7–14.1)	0.639 (0.295–1.383)		51.8	2.308 (0.876–6.080)	
UK	–	–		–	–	
Hepatectomy			0.631			0.587
No	9.7 (4.9–14.5)	1.000		59.5	1.000	
Yes	9.0 (2.9–15.1)	0.823 (0.371–1.826)		73.6	0.709 (0.205–2.451)	
Times of previous TACE		0.963 (0.722–1.286)^#^	0.800		1.537 (1.100–2.149)^#^	**0.012**
0	9.7 (5.8–13.6)	–		87.8	–	
1	11.4 (5.9–16.9)	–		59.3	–	
2	9.2 (3.0–15.5)	–		67.3	–	
3	5.1 (incalculable)	–		62.5	–	
>3	9.0 (0.0–19.0)	–		24.3	–	
Previous treatment lines		0.675 (0.302–1.511)^#^	0.339		1.512 (0.656–3.482)^#^	0.332
First-line	9.7 (5.7–13.6)	–		63.9	–	
Second-line	10.0 (2.4–17.5)	–		42.1	–	
>Second-line	incalculable	–		incalculable	–	
Times of TACE, No. (%)			0.111			0.095
≤3	9.7 (5.5–13.9)	1.000		61.0	1.000	
>3	11.0 (7.7–14.3)	0.479 (0.193–1.185)		77.8	0.281 (0.063–1.245)	
Timing of camrelizumab administration, No. (%)			0.222			0.159
Before TACE	10.0 (incalculable)	1.000		60.0	1.000	
After TACE	9.2 (5.1–13.3)	3.491 (0.470–25.920)		62.4	0.345 (0.078–1.515)	
Treatment cycle of camrelizumab			0.763			0.551
Q2W	9.0 (incalculable)	1.000		incalculable	1.000	
Q3W	9.7 (5.7–13.6)	0.801 (0.189–3.389)		60.4	21.574 (0.001–517,391.446)	
Cycles of camrelizumab, No. (%)		0.503 (0.320–0.792) ^#^	**0.003**		0.401 (0.221–0.729) ^#^	**0.003**
≤2	6.5 (1.6–11.5)	–		60.0	–	
3–4	3.9 (2.1–5.8)	–		19.1	–	
>4	11.4 (10.5–12.3)	–		82.4	–	
Interval between TACE and camrelizumab administration		1.702 (1.039–2.790)^#^	**0.035**		2.542 (1.398–4.620)^#^	**0.002**
Within 7 days	11.0 (9.0–13.0)	–		67.5	–	
Within 8 to 14 days	6.7 (incalculable)	–		66.7	–	
Within 15 to 28 days	4.2 (3.7–4.7)	–		0.0	–	
Treatment regimen			0.824			0.460
Monotherapy of camrelizumab	10.0 (3.6–16.3)	1.000		58.6	1.000	
Combination therapy with TKI	9.7 (7.1–12.3)	1.082 (0.539–2.171)		64.0	0.704 (0.277–1.787)	

PFS, progression-free survival; OS, overall survival; HR, hazard ratio; CI, confidence interval; HBV, hepatitis B virus; ECOG PS, Eastern Cooperative Oncology Group Performance Status; BCLC, Barcelona Clinic Liver Cancer; CNLC, China liver cancer; AFP, alpha-fetoprotein; UK, unknown; TACE, transarterial chemoembolization; Q2W, every 2 weeks; Q3W, every 3 weeks; TKI, tyrosine kinase inhibitors.

*Median OS was incalculable, thus 1-year OS rate was used; ^#^The variables were regarded as ordinal categorical variables instead of polytomous variable. The bold values indicate the comparison with statistical significance.

**Figure 4:**
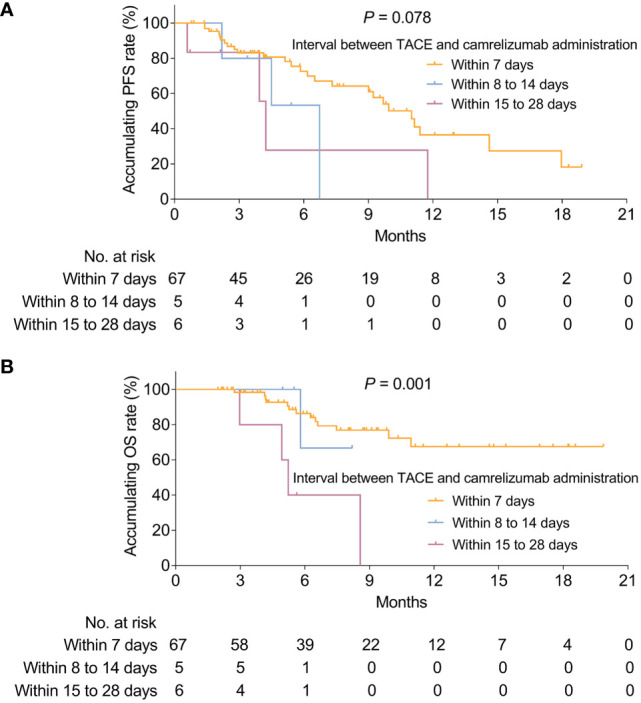
Correlation of timing between camrelizumab administration and TACE with survival. Correlation of timing between camrelizumab administration and TACE with PFS **(A)** and OS **(B)** in HCC patients receiving camrelizumab plus TACE. HCC, hepatocellular carcinoma; TACE, transarterial chemoembolization; PFS, progression-free survival; OS, overall survival.

Based on the findings from the subgroup analysis of PFS and OS, the association of the interval between camrelizumab administration and TACE with survival was subsequently determined by KM curve and log-rank tests, which indicated that the interval between camrelizumab administration and TACE was not correlated with PFS (*P* = 0.078, **Figure 4A**). However, the OS of those patients with a different interval between camrelizumab administration and TACE was varied (*P* = 0.001, **Figure 4B**). In detail, patients with camrelizumab administration within 15 to 28 days of the perioperative period of the TACE operation had the lowest accumulating OS rate, followed by those with camrelizumab administration within 8 to 14 days of the perioperative period of the TACE operation, and the highest in those with camrelizumab administration within 7 days of the perioperative period of the TACE operation. Apart from that, the baseline features between patients who were with and without TACE refractory were also compared; their baseline features were almost the same, except that the AFP level was higher in HCC patients without refractory to the TACE treatment (*P* = 0.038, **Supplementary Table 1**). However, the ORR (*P* = 0.610, **Supplementary Figure 3A**), PFS (*P* = 0.809, **Supplementary Figure 3B**), and OS (*P* = 0.250, **Supplementary Figure 3C**) were similar between these two groups.

### Independent factors predicting the survival

To evaluate the independent factors predicting PFS and OS, the multivariate Cox’s proportional hazards regression analysis was performed, which displayed that more cycles of camrelizumab were an independent factor in predicting the longer PFS (HR: 0.645, 95% CI: 0.243–1.708, *P* = 0.002), whereas a longer interval between camrelizumab administration and TACE was independently associated with pejorative PFS (HR: 1.873, 95% CI: 1.506–3.322, *P* = 0.032, **Table 4**). Additionally, the presence of vascular invasion (HR: 9.030, 95% CI: 2.355–34.629, *P* = 0.001), more times of previous TACE (HR: 1.618, 95% CI: 1.088–2.407, *P* = 0.018) were independent factors in predicting unfavorable OS.

**Table 4:** Factors affecting PFS and OS by multivariate Cox’s proportional hazards regression analysis.

Items	PFS		OS	
	*P*-value	HR (95% CI)	*P*-value	HR (95% CI)
Vascular invasion (yes vs. no)	0.147	1.775 (0.818–3.852)	**0.001**	9.030 (2.355–34.629)
Higher CNLC stage	0.950	1.016 (0.619–1.667)	0.384	1.478 (0.613–3.560)
AFP (≥400 ng/ml vs. <400 ng/ml)	0.274	0.638 (0.285–1.427)	0.079	2.636 (0.895–7.762)
More times of previous TACE	0.389	0.851 (0.590–1.228)	**0.018**	1.618 (1.088–2.407)
More times of TACE	0.377	0.645 (0.243–1.708)	0.107	0.233 (0.040–1.368)
More cycles of camrelizumab	**0.002**	0.415 (0.240–0.718)	0.070	0.484 (0.220–1.062)
Longer interval between TACE and camrelizumab administration	**0.032**	1.873 (1.056–3.322)	0.097	1.811 (0.898–3.654)

PFS, progression-free survival; OS, overall survival; HR, hazard ratio; CI, confidence interval; CNLC, China liver cancer; AFP, alpha-fetoprotein; TACE, transarterial chemoembolization. The bold values indicate the comparison with statistical significance.

### AEs

The incidence of total AEs was 90.1%. Besides, most AEs were grade 1 (20.8%), grade 2 (28.7%), and grade 3 (37.6%), while only three (3.0%) patients caused grade 4 AEs (**Table 5**). Concerning the hematologic AEs, the overall incidence was 90.1% and the most common hematologic AEs included transaminase elevation (60.4%), thrombocytopenia (57.4%), hypoalbuminemia (54.5%), hyperbilirubinemia (47.5%), leukopenia (42.6%), neutropenia (40.6%), anemia (39.6%), albuminuria (26.7%), and creatinine elevation (3.0%). Regarding the non-hematologic AEs, with an overall incidence of 28.7%, and the most common non-hematologic AEs were immune-related AEs (7.9%), rash (6.9%), reactive cutaneous capillary endothelial proliferation (RCCEP) (5.9%), fever (5.0%), pain (5.0%), fatigue (5.0%), loss of appetite (4.0%), hand-foot syndrome (1.0%), as well as nausea and vomiting (1.0%).

**Table 5:** AEs.

Items	Total	Grade 1	Grade 2	Grade 3	Grade 4
**Total AEs, No. (%)**	91 (90.1)	21 (20.8)	29 (28.7)	38 (37.6)	3 (3.0)
**Hematologic AEs, No. (%)**	91 (90.1)	24 (23.8)	27 (26.7)	37 (36.6)	3 (3.0)
Transaminase elevation, No. (%)	61 (60.4)	26 (25.7)	18 (17.8)	17 (16.8)	0 (0.0)
Thrombocytopenia, No. (%)	58 (57.4)	24 (23.8)	15 (14.9)	17 (16.8)	2 (2.0)
Hypoalbuminemia, No. (%)	55 (54.5)	35 (34.7)	19 (18.8)	1 (1.0)	0 (0.0)
Hyperbilirubinemia, No. (%)	48 (47.5)	28 (27.7)	16 (15.8)	4 (4.0)	0 (0.0)
Leukopenia, No. (%)	43 (42.6)	12 (11.9)	25 (24.8)	6 (5.9)	0 (0.0)
Neutropenia, No. (%)	41 (40.6)	17 (16.8)	12 (11.9)	11 (10.9)	1 (1.0)
Anemia, No. (%)	40 (39.6)	30 (29.7)	9 (8.9)	1 (1.0)	0 (0.0)
Albuminuria, No. (%)	27 (26.7)	17 (16.8)	8 (7.9)	2 (2.0)	0 (0.0)
Creatinine elevation, No. (%)	3 (3.0)	2 (2.0)	1 (1.0)	0 (0.0)	0 (0.0)
**Non-hematologic AEs, No. (%)**	29 (28.7)	16 (15.8)	10 (9.9)	2 (2.0)	0 (0.0)
Immune-related AEs	8 (7.9)	5 (5.0)	2 (2.0)	1 (1.0)	0 (0.0)
Rash, No. (%)	7 (6.9)	6 (5.9)	1 (1.0)	0 (0.0)	0 (0.0)
RCCEP, No. (%)	6 (5.9)	3 (3.0)	3 (3.0)	0 (0.0)	0 (0.0)
Fever, No. (%)	5 (5.0)	4 (4.0)	1 (1.0)	0 (0.0)	0 (0.0)
Pain, No. (%)	5 (5.0)	4 (4.0)	1 (1.0)	0 (0.0)	0 (0.0)
Fatigue, No. (%)	5 (5.0)	3 (3.0)	1 (1.0)	1 (1.0)	0 (0.0)
Loss of appetite, No. (%)	4 (4.0)	3 (3.0)	0 (0.0)	1 (1.0)	0 (0.0)
Hand foot syndrome, No. (%)	1 (1.0)	0 (0.0)	1 (1.0)	0 (0.0)	0 (0.0)
Nausea and vomiting, No. (%)	1 (1.0)	0 (0.0)	1 (1.0)	0 (0.0)	0 (0.0)

AEs, adverse events; RCCEP, reactive cutaneous capillary endothelial proliferation.

## Discussion

TACE has been shown to have anti-tumor efficacy. It is recognized as one of the most common nonsurgical treatments for patients with intermediate to advanced HCC. At the same time, it might simultaneously lead to the post-therapy neoangiogenetic reaction or induce hypoxia, which further leads to increased expression of programmed death-1 ligand (PD-L1); survival of HCC patients is still undesirable (7–9, 19, 20). For instance, one study indicated that TACE might upregulate the pro-inflammatory pathways; meanwhile, it was associated with the low intratumoral density of immune-exhausted effector cytotoxic and Tregs and further regulated the microenvironment of HCC (20). Another study exhibited that TACE might be involved in regulating post-therapy neoangiogenetic reactions *via* altering VEGF expression in HCC patients (21). The emergence of novel drugs such as PD-1 inhibitors mechanically leads to the possibility of combination therapy with TACE, whose combination has shown a certain efficacy in patients with HCC (11). Thus, to investigate the efficacy of TACE plus PD-1 inhibitor in HCC patients in a real-world study, we conducted a prospective, real-world study with a large sample size (including 101 patients with intermediate to advanced HCC). Meanwhile, all patients received camrelizumab-based treatment within 30 days of the TACE operation. We found that: 1) the ORR and DCR were 57.7% and 87.2%, respectively. In addition, the median PFS was 9.7 months, and the OS was NR; 2) the presence of vascular invasion was not associated with the ORR, DCR, or PFS, whereas it correlated with unfavorable OS; the longer interval between camrelizumab administration and TACE was related to the unsatisfying OS; more cycles of camrelizumab correlated with satisfying PFS and OS; the timing of camrelizumab administration (before and after TACE) was not associated with the PFS and OS; and 3) the safety profile of patients with advanced HCC treated with camrelizumab plus TACE was acceptable and manageable.

Of note is the population in this study: Differing from the etiopathology of other countries in the world, the prevalence of risk factors related to the etiopathology of HCC, such as hepatitis B virus infection in China, is high, which results in approximately 50% of newly diagnosed HCC, as well as HCC-caused deaths, being from China, and they are characterized by more aggravating disease features at diagnosis, such as advanced stage (2–5). For instance, one epidemiological study in eastern China disclosed that the rate of hepatitis B virus infection reached 87.5% (22). In line with previous studies, we found that the rate of hepatitis B virus was 74.3% in this study. Therefore, more attention should be paid to the patients with HCC in China regarding their treatment. Thus, we enrolled 101 patients with intermediate-to-advanced HCC in China in this study. Meanwhile, we applied camrelizumab plus TACE to treat these patients and evaluated the efficacy and safety of camrelizumab plus TACE in treating patients with intermediate to advanced HCC in China.

TACE has been recognized as one of the most common treatment modalities for patients with intermediate to advanced HCC and has shown specific efficacy in those patients (23–25). For instance, one study showed that treatment with TACE in advanced HCC patients resulted in an ORR of 31.8% and a median PFS of 54 days (about 2 months) (25). Another study exhibited an ORR of 32.6% and a DCR of 82.6%, as well as a median PFS of 5.5 months after treating advanced HCC patients with TACE alone (24). In our study, the ORR was numerically higher than in previous studies (57.7% vs. 31.8%–32.6%), and the PFS was also numerically higher than previous studies (18.1 months vs. 5.5 months). The possible reason might be as follows: further TACE might lead to hypoxia, which further leads to increased PD-L1 expression, thus causing undesirable survival in HCC patients. However, the combination of PD-1 inhibitors could inhibit the linkage of PD-1 and PD-L1, which could further synergize with TACE and achieve better clinical efficacy (7–9, 19, 20).

As mentioned above, the combination of TACE with other modalities (such as TKI with or without a PD-1 inhibitor) has shown promising clinical efficacy. For instance, TACE plus sorafenib achieves a median OS of 1.55 years with a 5-year OS rate of 10.7% in HCC patients (26). TACE plus sorafenib shows an OS of 12.77 months in advanced HCC patients (27). Another retrospective study disclosed that treatment with pembrolizumab plus TACE and lenvatinib achieved an ORR of 47.1%, a DCR of 70.0%, a median PFS of 9.2 months and an OS of 18.1 months (28). In our study, the PFS was numerically higher in patients who received TACE plus TKI (9.7 months vs. 7 months), while it was similar in patients who received PD-1 inhibitor plus TACE and TKI (9.7 months vs. 9.2 months). The possible reason might be as follows: 1) TACE treatment not only causes a post-therapy neoangiogenetic reaction but also induces a low expression of Tregs *via* modulating the pro-inflammatory pathways. The combination of TACE with TKI only attenuated the neoangiogenetic reaction caused by TACE but could not affect the microenvironment. However, in our study, we combined TACE with a PD-1 inhibitor to treat HCC patients, and some patients also received TKI therapy. Therefore, in these patients, the effect of neoangiogenetic reaction and the occurrence of immune tolerance caused by TACE could be attenuated by the TKI and PD-1 inhibitor. They might achieve a better clinical outcome. Besides, the OS was numerically higher in patients who received TACE plus sorafenib compared with TACE plus TKI in our study. These phenomena might derive from the material of TACE (conventional or drug-eluting beads), the clinical features of patients, and the treatment regimen.

Beyond that, the combination of PD-1 inhibitor and TKI is also greatly interesting in the clinical field. For instance, the ORR and PFS are 46.0% and 9.3 months in unresectable hepatocellular carcinoma treated with lenvatinib plus pembrolizumab, respectively (29). Additionally, the RESCUE study disclosed an ORR of 34.3% and a PFS of 5.7 months in advanced HCC patients who received camrelizumab plus apatinib (30). Meanwhile, some cases have also been reported to respond to the combination of pembrolizumab plus sorafenib (31, 32). In our study, the ORR (57.7% vs. 34.3%–46.0%) and PFS (9.7 months vs. 5.7–9.3 months) were slightly higher compared to patients receiving PD-1 inhibitor plus TKI. This phenomenon could be explained as follows: 1) The combination of TACE, PD-1 inhibitor, and TKI might have a synergistic effect compared to the use of PD-1 inhibitor plus TKI only, thus achieving a better efficacy profile with the former one; and 2) In our study, only parts of patients received the TACE plus PD-1 inhibitor and TKI, while the remaining patients received TACE plus PD-1 inhibitor only. Therefore, the efficacy superiority of TACE plus PD-1 inhibitor and TKI in this study was small compared with PD-1 inhibitor plus TKI in other studies.

Apart from the main findings for efficacy, we also found some interesting discoveries for efficacy from the subgroup analysis and multivariate Cox’s regression analysis, which disclosed that the presence of vascular invasion was not associated with the ORR, DCR, or PFS, while correlated with unfavorable OS; a longer interval between camrelizumab administration and TACE was related to the unsatisfying OS; more cycles of camrelizumab were correlated with satisfactory PFS and OS; and the timing of camrelizumab administration (before and after TACE) was not associated with the PFS and OS. Possible explanations might be that: 1) Although vascular invasion in HCC patients is known to be related to pejorative survival in a wide range of studies, the occurrence of vascular invasion was not associated with the ORR, DCR, or PFS in our study, which is possible due to the relatively few HCC patients being concurrent with vascular invasion (only 42 patients) (33); 2) Previous studies have exhibited that the long-term interval between TACE and other treatment modalities might yield a worse survival compared to the short-term interval between these two treatment modalities such as radiotherapy (34). In our study, we also found that the longer interval between camrelizumab administration and TACE was related to the unsatisfactory OS, which might be due to a decrease in intratumoral density of Tregs by TACE, further leading to an immune tolerance microenvironment. Meanwhile, the longer interval after TACE indicated the more mature the immune tolerance microenvironment, which weakened the efficacy of camrelizumab. Thus, a longer interval between camrelizumab administration and TACE caused a worse efficacy profile (20); 3) The number of cycles of camrelizumab administration was inherently determined by the clinical assessment of the responses and tolerance of patients, which implied that only the patients who responded to camrelizumab with tolerable AEs were likely to receive more cycles of camrelizumab administration. Therefore, those patients with more cycles of camrelizumab administration had a good response to camrelizumab with a tolerable and safe profile, resulting in a prolonged survival. 4) The timing of camrelizumab administration did not affect the efficacy and safety, which indicated that both strategies (camrelizumab administrated before or after TACE) were effective and safe; therefore, clinicians might choose the most suitable treatment strategy depending on the physical conditions of patients.

In terms of the safety findings, previous studies of patients with intermediate to advanced HCC treated with PD-1 inhibitor plus TACE indicated that the most common adverse events included fever, skin reactions, fatigue, vomiting, hypertension, diarrhea, thrombocytopenia, elevated AST, elevated ALT, asthenia, decreased appetite, rash, and pruritus (12, 28). Consistent with previous studies, we found that the incidence of total AEs was 90.1%. Besides, most AEs were below grade 3, including transaminase elevation, thrombocytopenia, hypoalbuminemia, hyperbilirubinemia, leukopenia, neutropenia, anemia, and albuminuria. In contrast, grade 4 AEs only occurred in three HCC patients, including grade 4 thrombocytopenia in two patients, and one patient experienced grade 4 neutropenia. These data indicated that the safety profile of treating patients with intermediate to advanced HCC with camrelizumab plus TACE was acceptable and manageable. Also, these data remind the clinicians to closely monitor the occurrence of AEs during the treatment with camrelizumab plus TACE and dispose of them in time.

Some points should be clarified in this study, such as why 7 and 14 days are set as the cut-point intervals between the TACE and camrelizumab. According to the previous study, this issue might be explained as the PD-1/PD-L1 inhibitor treatment within 24 h after TACE might achieve an elevation trend in the rabbit model (35). Thus, theoretically, the camrelizumab should be applied to HCC patients as soon as possible after the TACE treatment. However, in clinical practice, considering the liver injury, the interval between TACE and camrelizumab should be set for at least 7 days. In particular, the liver function of HCC patients would recover spontaneously within 3–7 days after TACE. Therefore, to confirm that all patients are recovered in terms of their liver function, we set the interval as 7 days. Secondly, another cut-off time point is set as 14 days, which could be explained as that some patients might suffer from severe liver injury, which implies that they could not recover from the liver injury spontaneously, while they need extra medicine treatment for the liver injury; therefore, they need another 7 days for this treatment. In this study, the interval between TACE and camrelizumab might affect survival, implying that the shorter interval could lead to a prolonged OS profile. At the same time, more attention should be paid to the recovery of liver function in these HCC patients. Additionally, all patients receive the cTACE treatment. This is because the patients in this study mainly include those with huge and multi-focal lesions. The efficacy of DEB-TACE in these patients is unsatisfactory. Besides, the expense of DEB-TACE is also high. Therefore, no DEB-TACE was applied in this study. However, the efficacy of DEB-TACE combined with camrelizumab in HCC patients with huge and multi-focal lesions could be determined in further study.

Several limitations should not be neglected. First, this study was a single-armed study, which lacked a control group; Second, even though we found that the longer interval between camrelizumab administration and TACE was related to the unsatisfactory PFS and OS, due to the relatively small number of patients in the subgroup of timing between camrelizumab administration and TACE within 15 to 28 days and those within 8 to 14 days, this finding needed to be validated in further study; Third, 23 patients without eligible imaging assessment were excluded from the efficacy evaluation, which further reduced the number of patients available for efficacy analysis; Fourth, the short follow-up period resulted in a median OS that has not yet been reached, thus a long-term follow-up in the further studies was needed; Fifth, other outcomes (i.e., quality of life) were not analyzed in this study; Sixth, due to the prevalence of risk factors was associated with the etiopathology of HCC, such as the hepatitis B virus infection varied between HCC patients in China and other countries. Thus, geographical limitations might exist which might lead to this finding being unsuitable for HCC patients from other countries; Seventh, in this study, a large number of patients (47.5%) received the various TKI agents during the study period, which may affect the results of the present study to a certain extent.

To be conclusive, the camrelizumab plus TACE regimen is effective and safe, indicating its potential to serve as a promising treatment choice for patients with intermediate to advanced HCC.

## Data availability statement

The original contributions presented in the study are included in the article/**Supplementary Material**. Further inquiries can be directed to the corresponding authors.

## Ethics statement

The studies involving human participants were reviewed and approved by The Institutional Review Board of Jiangsu Cancer Hospital and Jiangsu Institute of Cancer Research and The Affiliated Cancer Hospital of Nanjing Medical University. The patients/participants provided their written informed consent to participate in this study.

## Author contributions

RY and QX made substantial contributions to the design of the present study. Data acquisition was performed by RY, QX, QW, QZ, WZ, CC, XH, HHJ, PL, HJ, YL, YJ, YJL, LC, WW, HX, XZ, and GY. Data analysis was performed by RY, QX, QW, QZ, WZ, HX, XZ, and GY. Data interpretation was performed by RY, QX, HX, XZ, and GY. HX, XZ, and GY critically revised the manuscript for important intellectual content. All authors approved the final version of the manuscript.

## Conflict of interest

The authors declare that the research was conducted in the absence of any commercial or financial relationships that could be construed as a potential conflict of interest.

## Publisher’s note

All claims expressed in this article are solely those of the authors and do not necessarily represent those of their affiliated organizations, or those of the publisher, the editors and the reviewers. Any product that may be evaluated in this article, or claim that may be made by its manufacturer, is not guaranteed or endorsed by the publisher.

## Supplementary material

Supplementary Figure 1 MRI images at multiple-time-points for a typical HCC patient with PR after the combination treatment. MRI, magnetic resonance imaging; HCC, hepatocellular carcinoma; PR, partial response.

Supplementary Figure 2 CT and MRI images at multiple-time-points for a typical HCC patient with PR after the combination treatment. CT, computerized tomography; MRI, magnetic resonance imaging; HCC, hepatocellular carcinoma; PR, partial response.

Supplementary Figure 3 Comparison of the treatment response and survival between patients with and without TACE refractory. Comparison of the ORR **(A)**, PFS **(B)**, and OS **(C)** between HCC patients with and without TACE refractory. TACE, transarterial chemoembolization; ORR, objective response rate; PFS, progression-free survival; OS, overall survival; HCC, hepatocellular carcinoma.

Supplementary Table 1 Clinical characteristics between HCC patients with or without TACE refractory.
