# Supplementary figures and images for: Efficacy and safety of camrelizumab plus transarterial chemoembolization in intermediate to advanced hepatocellular carcinoma patients: A prospective, multi-center, real-world study
Source: Front Oncol. 2022 Aug 2;12:816198. doi: 10.3389/fonc.2022.816198 (PMC9378838; doi:10.3389/fonc.2022.816198)

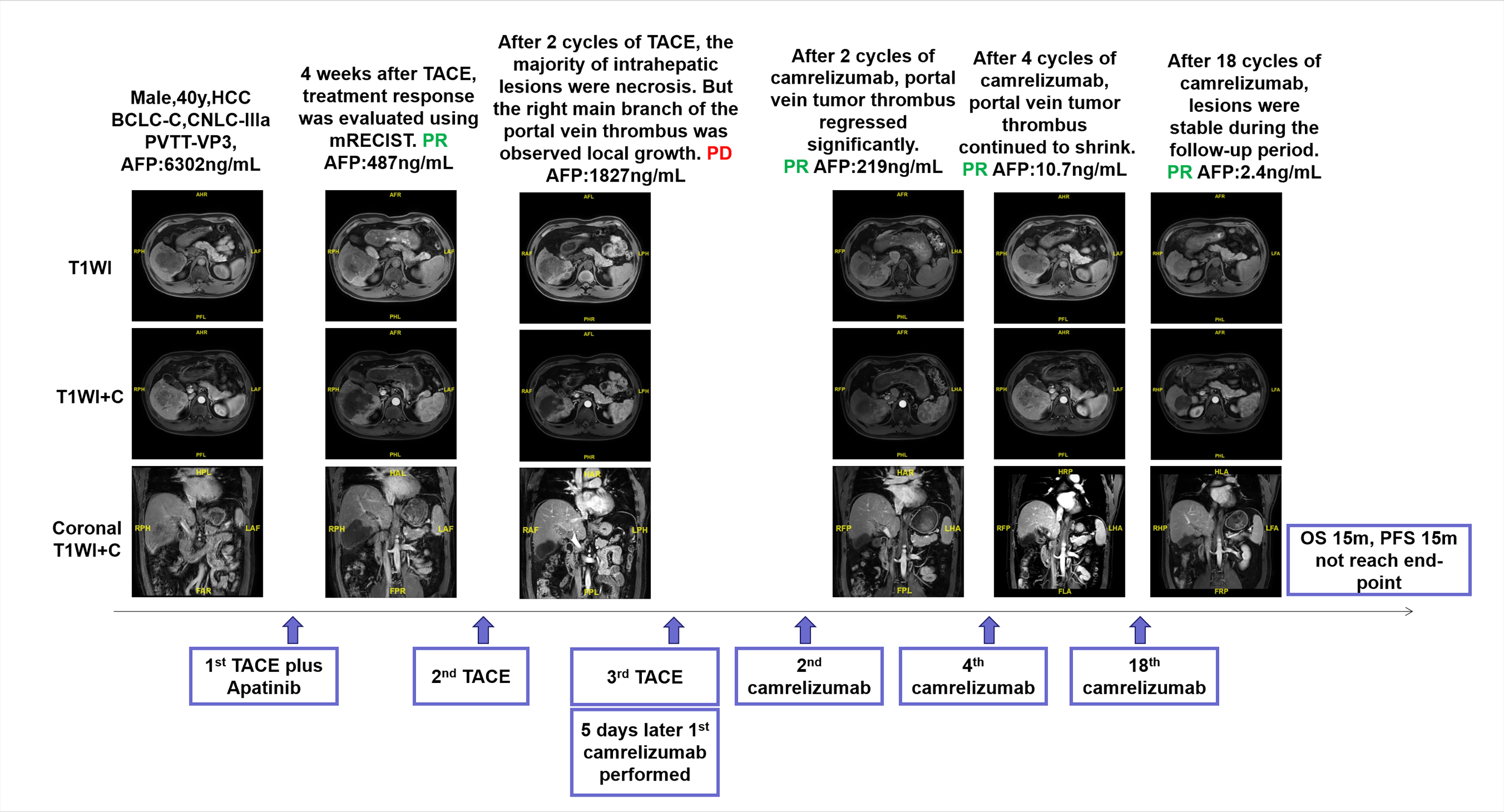

Supplement: Supplementary Figure 1 — MRI images at multiple-time-points for a typical HCC patient with PR after the combination treatment. MRI, magnetic resonance imaging; HCC, hepatocellular carcinoma; PR, partial response. [file Image_1.tif]

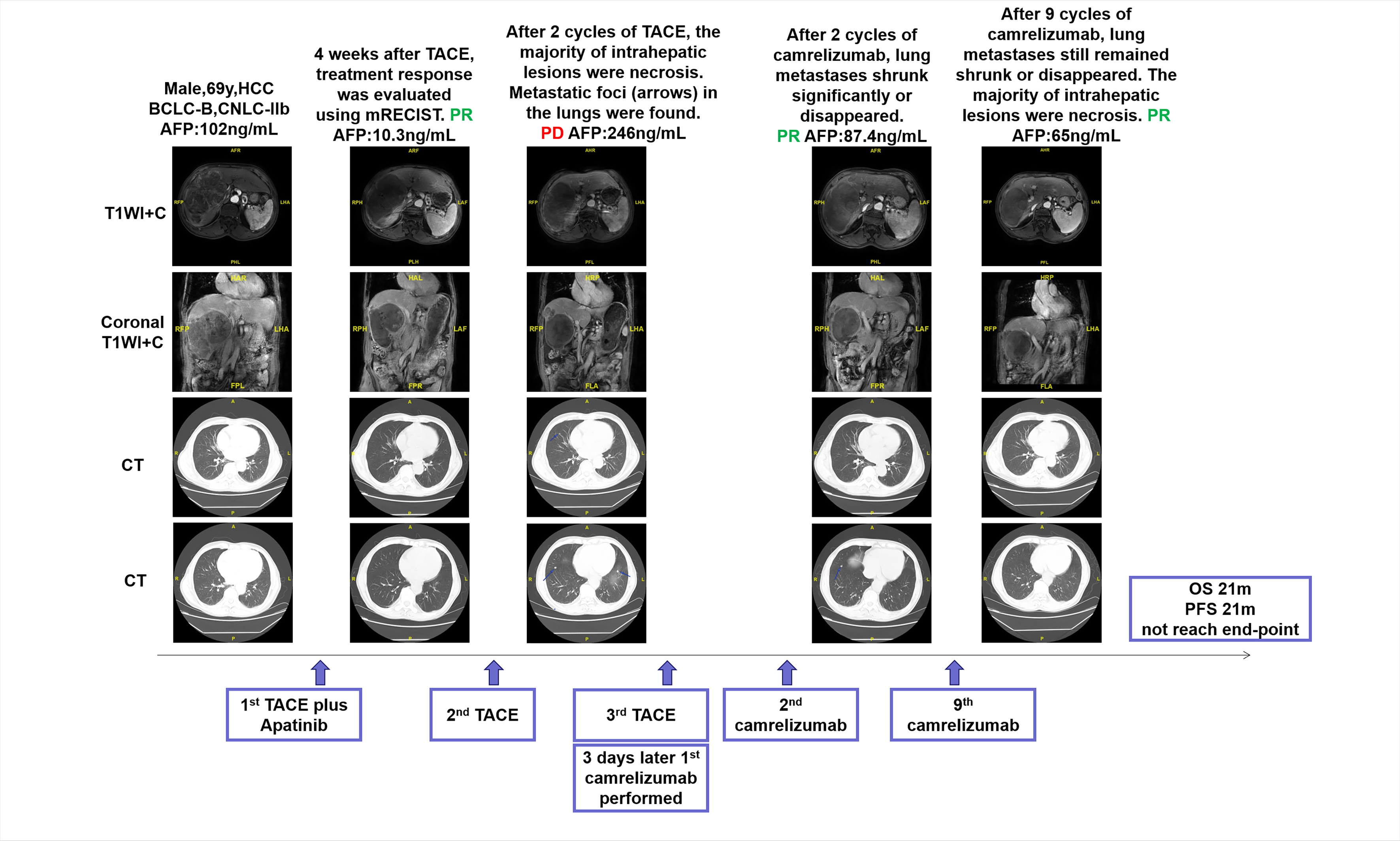

Supplement: Supplementary Figure 2 — CT and MRI images at multiple-time-points for a typical HCC patient with PR after the combination treatment. CT, computerized tomography; MRI, magnetic resonance imaging; HCC, hepatocellular carcinoma; PR, partial response. [file Image_2.tif]

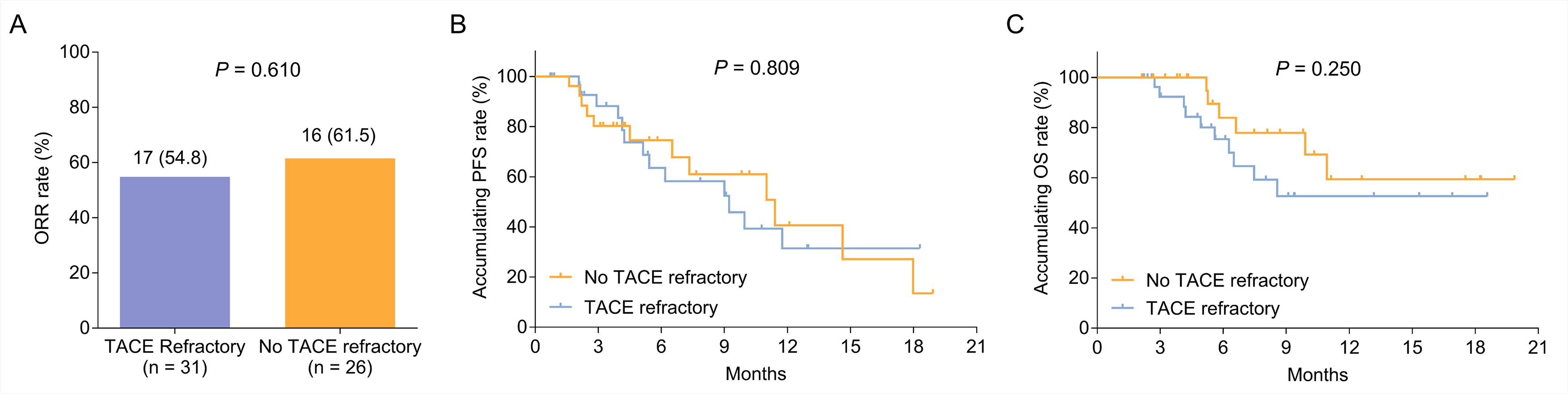

Supplement: Supplementary Figure 3 — Comparison of the treatment response and survival between patients with and without TACE refractory. Comparison of the ORR (A), PFS (B), and OS (C) between HCC patients with and without TACE refractory. TACE, transarterial chemoembolization; ORR, objective response rate; PFS, progression-free survival; OS, overall survival; HCC, hepatocellular carcinoma. [file Image_3.tif]
