# Supplementary material for: Efficacy and safety of camrelizumab plus transarterial chemoembolization in intermediate to advanced hepatocellular carcinoma patients: A prospective, multi-center, real-world study
Source: Front Oncol. 2022 Aug 2;12:816198. doi: 10.3389/fonc.2022.816198 (PMC9378838; doi:10.3389/fonc.2022.816198)
Supplement: Supplementary Table 1 — Clinical characteristics between HCC patients with or without TACE refractory. [file Table_1.docx]

Table 1. Clinical characteristics

| **Items** | HCC patients (N = 101) |
| --- | --- |
| **Demographic characteristics** |  |
| Age (years), mean±SD | 56.8±11.2 |
| Gender, No. (%) |  |
| Female | 12 (11.9) |
| Male | 89 (88.1) |
| **Disease characteristics** |  |
| HBV, No. (%) | 75 (74.3) |
| ECOG PS score, No. (%) |  |
| 0 | 26 (25.7) |
| 1 | 74 (73.3) |
| 2 | 1 (1.0) |
| Child-Pugh class, No. (%) |  |
| A | 72 (71.3) |
| B | 29 (28.7) |
| Extrahepatic metastasis, No. (%) | 56 (55.4) |
| Vascular invasion, No. (%) | 42 (41.6) |
| BCLC stage, No. (%) |  |
| B | 29 (28.7) |
| C | 72 (71.3) |
| CNLC stage, No. (%) |  |
| Ib | 1 (1.0) |
| IIa | 1 (1.0) |
| IIb | 16 (15.8) |
| IIIa | 26 (25.8) |
| IIIb | 56 (55.4) |
| UK | 1 (1.0) |
| AFP (ng/mL), No. (%) |  |
| < 400 | 57 (56.4) |
| ≥ 400 | 39 (38.6) |
| UK | 5 (5.0) |
| **Treatment history** |  |
| Hepatectomy, No. (%) | 27 (26.7) |
| Times of previous TACE, No. (%) |  |
| 0 | 29 (28.7) |
| 1 | 32 (31.7) |
| 2 | 16 (15.8) |
| 3 | 7 (6.9) |
| > 3 | 17 (16.8) |
| Refectory to TACE in patients with TACE treatment history, No. (%) | |
| No | 26 (25.7) |
| Yes | 31 (30.7) |
| Previous treatment lines, No. (%) |  |
| First-line | 82 (81.2) |
| Second-line | 17 (16.8) |
| > Second-line | 2 (2.0) |
| **Treatment in the study** |  |
| Times of TACE, No. (%) |  |
| ≤ 3 | 88 (87.1) |
| > 3 | 13 (12.9) |
| Timing of camrelizumab administration, No. (%) |  |
| Before TACE | 9 (8.9) |
| After TACE | 92 (91.1) |
| Treatment cycle of camrelizumab |  |
| Q2W | 2 (2.0) |
| Q3W | 99 (98.0) |
| Cycles of camrelizumab, No. (%) |  |
| ≤ 2 | 12 (11.9) |
| 3-4 | 33 (32.7) |
| > 4 | 56 (55.4) |
| Interval between TACE and camrelizumab administration, No. (%) | |
| Within 7 days | 84 (83.2) |
| Within 8 to 14 days | 9 (8.9) |
| Within 15 to 28 days | 8 (7.9) |
| Treatment regimen, No. (%) |  |
| Monotherapy of camrelizumab | 53 (52.5) |
| Combination therapy with TKI | 48 (47.5) |
| Apatinib | 27 (26.7) |
| Lenvatinib | 10 (9.9) |
| Sorafenib | 4 (4.0) |
| Anlotinib | 4 (4.0) |
| Regorafenib | 3 (3.0) |

HCC, hepatocellular carcinoma; SD, standard deviation; HBV, hepatitis B virus; ECOG PS, Eastern Cooperative Oncology Group Performance Status; BCLC, Barcelona Clinic Liver Cancer; CNLC, China liver cancer; UK, unknown; AFP, alpha-fetoprotein; TACE, transarterial chemoembolization; Q2W, every 2 weeks; Q3W, every 3 weeks; TKI, tyrosine kinase inhibitors.
